# Baseline Factors Predictive of SLT Response: A Prospective Study

**DOI:** 10.1155/2012/642869

**Published:** 2012-07-31

**Authors:** Robin Bruen, Mark R. Lesk, Paul Harasymowycz

**Affiliations:** ^1^Department of Ophthalmology, University of Montreal, Montreal, QC, Canada; ^2^Maisonneuve-Rosemont Hospital Research Centre, Montreal, QC, Canada

## Abstract

*Purpose*. To study the response to Selective Laser Trabeculoplasty (SLT) according to baseline medical treatment, angle pigmentation, age, diagnosis (open-angle glaucoma or ocular hypertension), and baseline intraocular pressure (IOP). *Methods*. 74 eyes of 74 patients were enrolled in this study. Baseline characteristics were recorded for each patient. IOP in the treated and fellow eyes was measured at baseline, and 1 month, 6 months, and 12 months following SLT. IOP changes in the different groups were compared using two-way ANOVA and Pearson's correlation. *Results*. The mean age of our cohort was 71 ± 10 years. The mean baseline IOP was 21.5 ± 5 mmHg, and the mean change in IOP from baseline in the treated eye at one year was −4.67 ± 3.40 mmHg. Higher baseline IOP was highly correlated with greater absolute IOP decrease. Prostaglandin analogue use at baseline was shown to be associated with a statistically decreased IOP-lowering response following SLT when corrected for baseline IOP. No significant differences in IOP response were found when comparing groups stratified for age, angle pigmentation, phakic status, gender, or diagnosis. *Discussion*. The results of this study confirm the finding that higher baseline IOP is a predictor of greater IOP response following SLT, and that pretreatment with prostaglandin analogue therapy is associated with a decreased IOP-lowering response following SLT. The study is limited by the small number of eyes with data available for complete case analysis.

## 1. Introduction

The introduction of selective laser trabeculoplasty (SLT) by Latina and colleagues in 1995 [1] has provided ophthalmologists with an additional modality for the treatment of open-angle glaucoma (OAG) and ocular hypertension (OHT). The results of the recent SLT/Med study [2] support earlier evidence [3–6] that primary therapy with SLT can lower intraocular pressure (IOP) as much as certain medical therapies. In clinical practice, however, SLT is often used as adjunctive therapy with topical medications.

Latina first examined the interaction between SLT and different classes of glaucoma medications in 2004 [7] and found an increased rate of nonresponders among patients treated with prostaglandin analogues compared to other topical glaucoma treatments. Subsequently, three retrospective case series have addressed the question directly with inconsistent findings. Scherer [8] reported a greater IOP decrease following SLT in patients on concomitant prostaglandin analogue therapy, while Singh et al. [9] did not find any relationship, and most recently Kara et al. [10] showed a decreased response to SLT in patients on prostaglandin analogue versus timolol and dorzolamide therapy.

Several distinct mechanisms of action have been theorized to cause the decrease in IOP following laser trabeculoplasty. The cellular theory holds that the phagocytic activity of macrophages in response to trabecular meshwork (TM) damage and cellular necrosis results in a clearing of debris and improved outflow [11]. The mechanical theory, which was especially thought to play a role in argon laser trabeculoplasty (ALT), holds that collagen shrinkage and scarring from the laser allow better drainage of aqueous humour [12]. A third theorized mechanism of action for SLT-induced IOP lowering is the alteration of the equilibrium between a family of proteolytic enzymes, known as matrix metalloproteinases (MMPs), and their inhibitors, known as tissue inhibitors of metalloproteinases (TIMPs) [13, 14].

The IOP-lowering mechanisms of prostaglandin analogues have also been extensively studied. They have been shown to be mediated by an effect on the uveoscleral [15] and the trabecular outflow pathways [16], and their mechanism of action has also implicated the equilibrium of MMPs and TIMPs [16]. Commonalities between the IOP-lowering mechanisms of prostaglandin analogues and SLT have been demonstrated by *in vitro* studies by Alvarado and colleagues [17], which have shown that both SLT and prostaglandin analogues induce increased permeability of cultured trabecular meshwork cells via intercellular junction disassembly.

SLT has been shown to decrease IOP not only in the treated eye, but also in the fellow eye [18], suggesting that at least some of the IOP-lowering effect of SLT is based on cellular or MMP-mediated mechanisms.

This paper presents the results of a prospective nonrandomized interventional cohort study undertaken at our institution to assess the response to SLT according to baseline medical treatment, angle pigmentation, age, type of open-angle glaucoma or ocular hypertension, and baseline intraocular pressure.

## 2. Patients and Methods

The Research Ethics Board at the University of Montreal approved this prospective, interventional cohort study, which adhered to the tenets of the declaration of Helsinki. All patients were recruited from the clinics of two glaucoma specialists, PH and ML, at the Montreal Glaucoma Institute and Maisonneuve-Rosemont Hospital. Inclusion criteria included patients with a diagnosis of either OAG or OHT. Informed consent was obtained from all participants. In total, 74 eyes (30 OD 44 OS) of 74 patients were enrolled. In cases where both eyes were treated with SLT during the study period, only the left eye was counted. In one case, the left eye had been previously treated with SLT, so the results from the right eye were included.

Baseline characteristics of all patients were recorded, including age, gender, diagnosis (OAG or OHT), baseline IOP, history of prior ALT treatment, and the number and the type of medications being taken. In addition, angle pigmentation was evaluated using standardized photographs taken at the time of the procedure.

A Coherent Selecta 7000 laser (Coherent, Inc., Palo Alto, CA) was used to perform SLT over 360 degrees, with approximately 60 nonoverlapping applications in each eye. The initial intensity used was 0.8 mJ, but the energy was titrated up and down in 0.1 mJ increments in order to achieve occasional “champagne bubbles” during the treatment.

IOP was measured in both eyes at baseline, and 1 month, 6 months, and 12 months after SLT treatment. The outcome measures were change in IOP compared to baseline, and change in IOP compared to the fellow eye at each time point. However, if SLT was performed on the fellow eye during the study period, or if the medical therapy of the fellow eye changed during the study period, then the data comparing IOP in the SLT-treated eye to that of the fellow eye was not included in the analysis.

The data was analyzed using SPSS statistical software (version 15) using ANOVA for repeated measures, and Pearson's correlation. Time was used as a within-factors effect, and several baseline characteristics were used, one at a time, as between-factors effects: baseline IOP, age, sex, degree of angle pigmentation, phakic status, diagnosis (OAG or OHT), prior argon laser trabeculoplasty (ALT) treatment status, and type and number of medications.

Given the known diurnal variation in IOP, and assuming this diurnal variation is similar in both eyes, the analysis of the effect of the different baseline factors was performed using both the baseline IOP in the treated eye and the IOP in the fellow eye at each time point as a control. For the analysis using baseline IOP in the treated eye as a control, only eyes with IOP measurements at each time point, or complete case data, were included. For the analysis using the fellow eye IOP at each time point as a control, only eyes for which there were IOP measurements in both eyes at each time point were used. It was assumed that there was random attrition of the data over time.

## 3. Results

The mean age of our subjects subjects was 71 ± 10 years. Sixty-two patients (83.8%) had a vertical cup-to-disk ratio of at least 0.7. Mean IOP was 21.5 ± 5 mmHg, and patients were taking an average of 2 ± 1 topical medications for IOP control at the onset of the study. Subgroup analysis was performed on the 42 patients for whom there was complete case data.

In our sample, the mean change in IOP from baseline in the treated eye at one year was −4.67 ± 3.40 as shown in Table 1. At every time point, there was a greater mean change in IOP in the SLT-treated eye from baseline than when this change was controlled for IOP changes in the fellow eye, providing validation for using the fellow eye IOP as a control.

Baseline IOP in the SLT-treated eye was found to be markedly predictive of IOP change across time in the same eye, such that eyes with higher baseline IOPs tended to have greater IOP reduction. Stratifying the eyes according to baseline IOP percentiles, there was a statistically significant difference in IOP decrease at the different time points between groups above and below the 50th percentile of 20 mmHg (*P* < 0.0001) (−6.37 ± 2.94 mmHg versus −2.10 ± 3.10 mmHg at one year, for baseline IOP above and below the 50th percentile, resp.), the 66th percentile of 23 mmHg (*P* < 0.003) (−7.25 ± 3.00 versus −3.37 ± 3.16 mmHg at one year for baseline IOP above and below the 66th percentile, resp.), and the 80th percentile of 25 mmHg (*P* < 0.001) (−5.44 ± 2.61 versus −1.63 ± 2.57 mmHg at one year, for baseline IOP above and below the 80th percentile, resp.).

This difference in IOP decrease over time of eyes above or below the given IOP percentiles was also noted when IOP in the SLT-treated eye was compared to the fellow eye at the different time points, although the relationship was less strong and not statistically significant for the 50th and 66th percentile comparisons. For the 50th percentile, the significance was *P* < 0.093 (−3.38 ± 2.94 versus −1.29 ± 2.88 mmHg at one year for eyes above and below the 50th percentile, resp.). For the 66th percentile, the significance was *P* < 0.06 (−4.86 ± 2.73 versus −1.57 ± 2.65 mmHg at one year for eyes above and below the 66th percentile, resp.). For the 80th percentile, the significance was *P* < 0.03 (−5.44 ± 2.62 versus −1.74 ± 2.81 mmHg at one year for eyes above and below the 80th percentile, resp.).

A total of 59 eyes (79.7%) from our sample of 74 eyes received medical treatment with prostaglandin analogues over the course of the study. Of the 42 patients for whom complete case data was available, 32 had been on prostaglandin analogue therapy at the beginning of our study. Using baseline IOP as a control, there was not a significant difference in IOP decrease over time between patients who had been taking prostaglandin analogues preoperatively, and those who had not. However, using fellow eye IOP as a control, there was a statistically significant difference in IOP decrease over time between patients who had been treated with prostaglandin analogues at baseline, and those who had not been treated with prostaglandin analogues at baseline.

For this latter analysis, complete case data including IOP of the fellow eye at each time point was available for 35 patients, 26 of whom had been treated with prostaglandin analogues prior to SLT. During the study period, medical therapy was unchanged in 22 out of the 35 patients in both eyes. Of the 13 patients with some modification of their medical therapy during the study period, 10 were in the prostaglandin group, 4 had additional drops added at 1 or 6 months due to inadequate IOP control (all in the prostaglandin group), 4 had drops temporarily removed following SLT then restarted at 1 or 6 months, and 5 had changes to their drops but not to the number of drops, including 2 who began taking prostaglandin analogues instead of aqueous suppressants and beta blockers.

**Figure 1:**
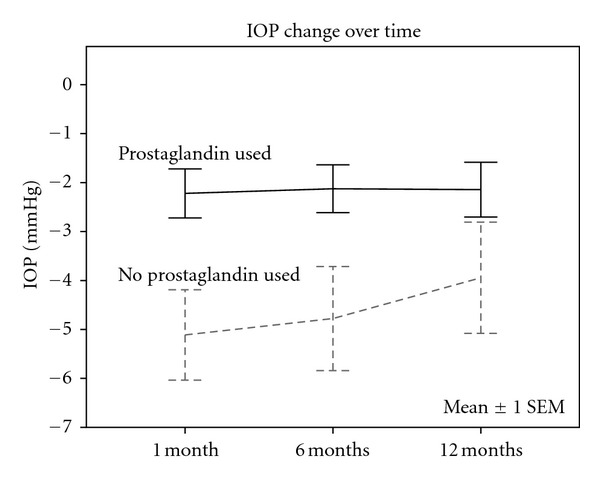
Graph comparing IOP change over time of patients treated with prostaglandin analogues to those treated with other medical treatments prior to SLT. The IOP change shown in the graph is the mean of the differences between the IOP of the treated and fellow eyes in both groups at each time point, and the IOP change has been controlled for baseline IOP.

No interaction between time and prostaglandin use was found (*P* = 0.606). Independently of time, a statistically significant difference in IOP decrease was found between eyes pretreated or not with prostaglandin analogues (*P* = 0.008), as shown in Table 2. This difference in IOP response to SLT between prostaglandin users and those not using prostaglandins remained significant even when the IOP change over time was corrected for baseline IOP (*P* = 0.006), as displayed in Figure 1.

There were 36 eyes with OAG and 6 eyes with OHT with complete case data to compare IOP response in the SLT-treated eye to baseline IOP in the same eye at each time point. There was a trend toward better IOP response in the OHT group, but this was not significant (*P* < 0.225). In a similar analysis, IOP in the SLT-treated eye was compared to IOP in the fellow eye at each time point. There were 30 eyes with OAG and 5 eyes with OHT for this analysis, and the results also showed a trend toward better IOP response at each time point for the OHT group, but the result was not significant (*P* < 0.182).

There were a total of 38 phakic and 4 pseudophakic eyes available for complete case analysis of the change in the SLT-treated eye compared to baseline. Overall, there was not a significant difference in IOP reduction over time between phakic and pseudophakic eyes (*P* < 0.225). However, there was a trend towards better response in phakic eyes compared to pseudophakic eyes at 1 and 6 months.

There were 5 eyes with prior ALT and 35 eyes without prior ALT available for the complete case analysis of the change of IOP in the SLT-treated eye compared to baseline IOP in the same eye. There was not a statistically significant difference between the groups (*P* < 0.629). Similarly, IOP in 29 eyes without prior ALT and 4 eyes with prior ALT was compared to IOP in the contralateral eye at each time point, and no statistically significant difference was found between groups (*P* < 0.785).

The degree of angle pigmentation of 42 eyes was available for complete case analysis of the SLT-treated eye IOP compared to baseline IOP in the same eye. The degree of pigmentation was 0+ in 3 eyes; 1+ in 11 eyes; 2+ in 12 eyes; 3+ in 12 eyes; and 4+ in 4 eyes. There was not a statistically significant difference in IOP change over time according to pigmentation. However, there was a trend towards less IOP response in the 0+ pigment eyes. A similar analysis comparing IOP in the SLT-treated eye to the IOP of the fellow eye at each time point was conducted. The pigmentation levels were 0+ in 2 eyes, 1+ in 11 eyes, 2+ in 8 eyes, 3+ in 10 eyes, and 4+ in 4 eyes. There was also not a significant difference in IOP response over time according to pigmentation levels (*P* < 0.127), but 0+ pigmentation eyes showed a trend to worse IOP response. Furthermore, no statistically significant difference in IOP response over time according to pigmentation level was found even when pigmentation levels were grouped combining 0+ and 1+, as well as 3+ and 4+ pigmentation levels into larger groups.

There were 6 eyes enrolled in the study with pseudoexfoliative glaucoma (PXFG), but there were only two for which IOP measurements in treated and fellow eyes were available at each time point. There was not a statistically significant difference (*P* = 0.231) in response to SLT between eyes with PXFG and those with other forms of OAG or OHT.

## 4. Discussion

The results of our study confirm the findings of some other authors that high baseline IOP is a predictor of IOP-lowering response after SLT [19–21]. In addition, our study found that gender, age, and degree of angle pigmentation did not predict response to SLT, which is consistent with much of the other literature on the subject. Although pigmentation did not influence IOP change over time in the present study sample, IOP spikes following SLT in some heavily pigmented eyes have previously been reported [22]. The trend to decreased IOP-lowering response over time in eyes with 0+ pigmentation levels might be due to decreased laser energy absorption or perhaps to increased difficulty in delivering the laser energy to the appropriate location on the TM.

Our study is limited by the small number of patients enrolled and by missing IOP data in the treated and fellow eyes, which limited the statistical power from our complete case analysis. In addition, the majority of patients in our sample were being treated with other glaucoma medications concurrently, making it difficult to eliminate confounding factors and drug interactions. The effect of premedication with other drops on SLT remains to be seen.

In this study, we observed a statistically significant weakening of the IOP-lowering response to SLT in eyes treated with prostaglandin analogue therapy, compared to prostaglandin naïve eyes. This diminished effect of SLT on these patients persisted even when we controlled for baseline IOP and was present at all time points. These findings are consistent with the findings of Latina and De leon [7], and Kara et al. [10].

Both prostaglandin analogues and selective laser trabeculoplasty result in intercellular junction disassembly, paracellular pathway widening, and increased conductivity in cultured Schlemm canal endothelial cells [23]. This observation provides a possible explanation for the empirically observed phenomenon of reduced marginal benefit of SLT for patients receiving prostaglandin analogues, which has been confirmed by the present study. Thus, the expected benefit of SLT appears to be greatest in prostaglandin naïve eyes with higher baseline IOP, and lower in prostaglandin-treated eyes with lower baseline IOP.

It remains to be seen whether patients initially treated with SLT would also benefit less from subsequent prostaglandin analogue therapy. If this is not the case, then it might be more beneficial to consider performing SLT as first-line therapy in some eyes, before instituting prostaglandin analogue therapy.

## Figures and Tables

**Table 1:** 

	Total study sample size	Sample with available data	Mean IOP change in mmHg	Standard deviation of IOP change in mmHg	Median of IOP change in mmHg	Maximum IOP decrease in mmHg	Maximum IOP increase in mmHg
Treated eye versus baseline at 1 month	74	70	−3.86	−3.93	−3.00	−17.00	5.00
Treated eye versus fellow eye at 1 month	74	70	−3.50	3.56	−3.50	−12.00	2.50
Treated eye versus baseline at 6 months	74	56	−3.80	5.03	−4.00	−16.00	15.50
Treated eye versus fellow eye at 6 months	74	47	−2.47	4.31	−2.00	−11.00	17.50
Treated eye versus baseline at 12 months	74	51	−4.67	3.40	−5.00	−17.00	3.00
Treated eye versus fellow eye at 12 months	74	43	−2.52	3.13	−3.00	−9.50	3.50

**Table 2:** 

	Prostaglandin used	Mean	Standard deviation	*N*
Change compared to contralateral eye 1 month after SLT	No	−5.111	2.770	9
	Yes	−2.221	2.558	26
	Total	−2.964	2.874	35

Change compared to contralateral eye 6 months after SLT	No	−4.778	3.193	9
	Yes	−2.125	2.487	26
	Total	−2.807	2.886	35

Change compared to contralateral eye 12 months after SLT	No	−3.944	3.404	9
	Yes	−2.144	2.848	26
	Total	−2.607	3.054	35
